# Determination of Volatile Organic Compounds and Endogenous Extracts and Study of Expression Patterns of *TPS* and *BSMT* in the Flowers of Seven *Lilium* Cultivars

**DOI:** 10.3390/molecules28247938

**Published:** 2023-12-05

**Authors:** Peng Zhang, Xiaoou Ma, Qian Zhang, Ziyu Guo, Junyi Hao, Zhixuan Zhang, Ming Sun, Yan Liu

**Affiliations:** State Key Laboratory of Efficient Production of Forest Resources, Beijing Key Laboratory of Ornamental Plants Germplasm Innovation and Molecular Breeding, National Engineering Research Center for Floriculture, Beijing Laboratory of Urban and Rural Ecological Environment, Key Laboratory of Genetics and Breeding in Forest Trees and Ornamental Plants of Ministry of Education, School of Landscape Architecture, Beijing Forestry University, Beijing 100083, China; gzyyndemailbox@163.com (P.Z.); xiaoou0629@163.com (X.M.); zhangqian3494@163.com (Q.Z.); gzybfu@bjfu.edu.cn (Z.G.); hjybeilin123456@163.com (J.H.); plantzzx@163.com (Z.Z.)

**Keywords:** *Lilium*, volatile organic compounds (VOCs), headspace solid-phase microextraction (HS-SPME), organic solvent extraction (OSE), gas chromatography–mass spectrometry (GC-MS), terpenoids, phenylpropanoids/benzenoids

## Abstract

Lily is one of the most important cut flowers in the world, with a rich floral fragrance. To further explore the fragrance emission mechanisms of lily cultivars, headspace solid-phase microextraction–gas chromatography–mass spectrometry (HS-SPME-GC-MS) and organic solvent extraction–gas chromatography–mass spectrometry (OSE-GC-MS) were used to unveil the volatile organic compounds (VOCs) and endogenous extracts of seven lily cultivars. Furthermore, real-time quantitative PCR (qRT-PCR) was used to determine the expression levels of two key genes (*TPS* and *BSMT*) related to the biosynthesis of monoterpenoids and methyl benzoate. The results show that forty-five VOCs were detected in the petals of seven lily cultivars, and the main compounds were monoterpenoids and phenylpropanoids/benzenoids. Dichloromethane was the best solvent for extracting the endogenous extracts of *Lilium* ‘Viviana’ petals and eighteen endogenous extracts were detected using dichloromethane to extract the petals of seven lily cultivars. Each compound’s emission ratio (natural logarithm of the ratio of VOC content to endogenous extract content) was calculated, and linear regression analyses between emission ratios and boiling points were conducted. Significant linear negative correlations existed between the emission ratios and boiling points of compounds, and the regression equations’ coefficients of determination (*R*^2^) were all greater than 0.7. *TPS* was expressed highly in ‘Viviana’, ‘Pink News’, and ‘Palazzo’, and *BSMT* was expressed highly in ‘Pink News’ and ‘Palazzo’. Correlation analyses between the gene expression levels and the monoterpenoids and methyl benzoate contents found that the *TPS* expression levels have strong positive correlations with monoterpenoids content, while no correlations were found between the expression levels of *BSMT* and the contents of methyl benzoate. This study lays the foundation for research on the release patterns of VOCs in the flowers of *Lilium*, and the breeding of lilies for their floral fragrance.

## 1. Introduction

*Lilium* spp. is an important bulbous plant of the Liliaceae family with high ornamental and economic value, and it occupies a prominent place in horticulture [1]. About one hundred wild species and ten thousand cultivars of lilies are found worldwide, mainly cultivated in the Netherlands, with a bulb production acreage of more than five thousand ha [2,3]. Lilies can be widely used at potted flowers, cut flowers and landscape applications, and are welcomed by many consumers [4].

Flower scents are composed of many volatile organic compounds (VOCs), including terpenoids, phenylpropanoids/benzenoids, fatty acid derivatives, and some compounds containing nitrogen or sulfur [5,6]. Flower scents can attract insects to pollinate flowers and play a role in plant defense [7]; simultaneously, they are decisive communication channels between plants and floral visitors [8]. Flower scents can also give people mental pleasure and are widely used in cosmetics, essential oils, foods and medical applications [9]. Lily petals can release multiple VOCs; monoterpenoids and phenylpropanoids/benzenoids are the main VOCs emitted from lily flowers. Four monoterpenoids (linalool, (E)-β-ocimene, 1,8-cineole and myrcene) and two phenylpropanoids/benzenoids (methyl benzoate and ethyl benzoate) are the most critical VOCs in most fragrant lilies [10,11]. The most commonly used cultivar for studying the VOCs (types and contents, molecular mechanisms of biosynthesis) of lily was *Lilium* ‘Siberia’ (Oriental hybrid groups); the flower of *Lilium* ‘Siberia’ contains plentiful cis-β-ocimene, linalool and methyl benzoate [7,12,13]. An important terpene synthase gene—*LiTPS2*—can promote the generation of monoterpene VOCs in *Lilium* ‘Siberia’ [14]. In addition, previous research confirmed that some transcription factors play a crucial role in the biosynthesis of terpenoids in *Lilium* ‘Siberia’. *LiMYB1*, *LiMYB305*, *LiMYB308* and *LiMYB330* in the MYB family [15,16], and *LibHLH22* and *LibHLH63* in the bHLH family [17] can promote the expressions of key terpenoid synthase genes, and promote the biosynthesis of monoterpenoids in *Lilium* ‘Siberia’. As regards the biosynthesis of phenylpropanoids/benzenoids in *Lilium*, a benzoic acid/salicylic acid carboxyl methyltransferase gene, *LiBSMT*, could promote the biosynthesis of methyl benzoate in *Lilium* ‘Yelloween’ [18]. However, further research should be carried out on *Lilium*’s mechanism of biosynthesizing other phenylpropanoids/benzenoids.

Most flower VOCs’ boiling points range from 150 to 350 °C, and are mainly present as a liquid or solution in the flower tissue. Due to the vapor pressure, only a portion can be emitted as a flower scent [19]. Most research on the release patterns of VOCs from flowers focuses only on the emitted volatile, with a significant portion of the VOCs kept in the floral tissue being often overlooked [20]. Endogenous compounds are the basis of substances’ volatilization, and the diversity of flower scents is caused by changes in endogenous extract content and the evaporation of VOCs; both are important when analyzing the release pattern of flower scents [21]. In this study, VOCs (or headspace VOCs) refers to the compounds emitted from flowers and collected by the headspace method, and endogenous extracts (or endogenous VOCs) are the compounds that remain in flower tissues and are extracted using organic solvents.

To discover VOCs in flowers usually involves employing headspace solid-phase microextraction (HS-SPME) combined with gas chromatography–mass spectrometry (GC-MS). Organic solvent extraction (OSE) combined with GC-MS is frequently used to find endogenous flower extracts. The solvent is essential in affecting the extraction of flower scents. Because of the different polarities of each solvent, there are differences in the solubility of flower scents in different solvents, which can affect the compounds in the extract. Some research on determining petal VOCs in *Lilium* has been published, and measurement methods have been effectively developed. However, little is known about the types and contents of flower endogenous extracts in *Lilium*.

To clarify the differences between VOCs and endogenous extracts and analyze the diversity of flower scents among different lily cultivars, HS-SPME-GC-MS and OSE-GC-MS were used to explore the VOCs and endogenous extracts of seven lily cultivars. We also compared the extraction effects of eight different solvents on flower endogenous extracts. The expression patterns of two key genes for floral fragrance biosynthesis (*TPS* [14] and *BSMT* [18]) in the flowers of seven lily cultivars during their peak flowering stage were analyzed. This study lays the foundation for understanding the precise rules of lily fragrance release, aroma type improvement, and flower fragrance molecular breeding.

## 2. Results

### 2.1. Determination Results of VOCs in Flowers of Seven Lily Cultivars

To calculate the RI of each floral fragrance compound, we first measured the retention times of n-alkanes (C7–C40); the results are shown in Table S1. We measured the VOCs of lily petals, and forty-five VOCs were detected in the petals of seven lily cultivars during the peak flowering stage; the results are shown in Table 1. From the perspective of VOC categories, these VOCs could be divided into six categories: terpenoids, phenylpropanoids/benzenoids, alcohols, ethers, aldehydes and esters. The number of terpenoids was the highest, and twenty-three terpenoids were detected, accounting for 51.11% of the total number of VOCs. Secondly, twelve phenylpropanoids/benzenoids were detected, accounting for 26.67% of the total VOCs. The number of alcohols, ethers, aldehydes and esters in all VOCs was relatively small, and ten compounds were detected, only accounting for 22.22% of the total VOCs. From the perspective of different lily cultivars, three Oriental hybrid cultivars and an OT hybrid cultivar were detected with more kinds of VOCs and higher contents. The OT lily ‘Palazzo’ contained more terpenoids and phenylpropanoids/benzenoids than other cultivars, while the Asiatic hybrid ‘Tresor’, LA hybrid ‘Trebbiano’ and OA hybrid ‘Avalon Sunset’ had fewer kinds of VOCs, with lower contents, compared to the other four lilies. Some VOCs such as β-pinene, β-ocimene, γ-terpinene, terpinolene, allo-ocimene, neo-allo-ocimene, geranylacetone, methyl benzoate, ethyl benzoate, creosol, butyl acrylate and methyl decanoate were detected in most of the seven cultivars. From the perspective of VOC contents, β-ocimene could be detected in multiple cultivars and became one of the most abundant VOCs among all components. Allo-ocimene and neo-allo-ocimene were also important VOCs, but their contents were not as high as that of β-ocimene. Although 1,8-cineole and linalool were only detected in a few cultivars, the contents of two VOCs were high in these cultivars. Methyl benzoate was the most common phenylpropanoid/benzenoid detected in lily petals with the highest content.

**Figure 1:**
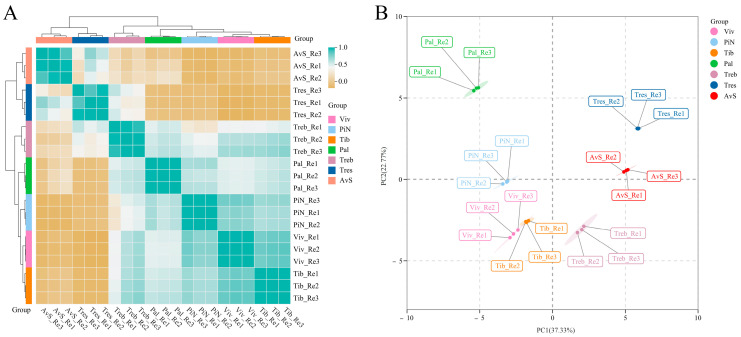
Sample correlation analysis (**A**) and PCA (**B**) of VOCs from the petals of seven lily cultivars. AvS: ‘Avalon Sunset’; Pal: ‘Palazzo’; PiN: ‘Pink News’; Tib: ‘Tiber’; Treb: ‘Trebbiano’; Tres: ‘Tresor’; Viv: ‘Viviana’. Three biological replicates were performed for VOC determination in each cultivar.

Sample correlation analysis and principal component analysis (PCA) were conducted using the contents of VOCs in the petals of seven lily cultivars. The results are shown in Figure 1—the results of determination of the VOCs in seven cultivars showed good repeatability. According to the result of sample correlation analysis, four richly fragrant lily cultivars (‘Palazzo’, ‘Pink News’, ‘Tiber’ and ‘Viviana’) and a lightly fragrant lily cultivar (‘Trebbiano’) clustered together. The clustering relationship of four richly fragrant cultivars (‘Palazzo’, ‘Pink News’, ‘Tiber’ and ‘Viviana’) was particularly close. Moreover, two other cultivars with almost no fragrance (‘Avalon Sunset’ and ‘Tresor’) were also clustered together. According to the PCA analysis results, four richly fragrant lily cultivars (‘Palazzo’, ‘Pink News’, ‘Tiber’ and ‘Viviana’) were separated from the other three lightly or almost non-fragrant lily cultivars (‘Trebbiano’, ‘Avalon Sunset’ and ‘Tresor’) as regards the first principal component (PC1 37.33%).

### 2.2. Comparison of Extraction Effects of Eight Solvents on Endogenous Extracts in Lily

We compared the extraction effects of eight solvents (ethyl acetate, dichloromethane, benzene, n-hexane, petroleum ether, methanol, ethanol and n-butanol) on endogenous extracts in the petals of *Lilium* ‘Viviana’ and performed GC-MS analysis. The types and contents of detected endogenous extracts are shown in Table S2. Based on the measurement results, we found that the standard (ethyl decanoate) could not be detected when using benzene as the solvent to extract petals, and any detected endogenous extracts could not be quantified. Therefore, Table S2 only displays the extraction results of the other seven solvents. Table S3 shows the peak areas of endogenous extracts obtained via petal extraction using benzene. 

From the results in Tables S2 and S3, we can see that the number of endogenous extracts detected by the OSE method in *Lilium* ‘Viviana’ decreased compared to VOCs. Although benzene could not detect the standard and quantify the endogenous extracts, it could extract twelve compounds, the most among the eight solvents. This was followed by dichloromethane (eleven compounds), petroleum ether (nine compounds), ethyl acetate (seven compounds) and n-hexane (seven compounds). The strong polarity solvents (methanol and ethanol) could only extract three endogenous compounds: creosol, 2-methoxy-4-vinylphenol (not detected in VOCs) and isoeugenol; the endogenous contents of these three compounds extracted using methanol and ethanol were much higher than those of other solvents. We extracted five compounds from the petals using n-butanol, which effect was slightly better than using methanol and ethanol. Benzene could extract more endogenous extracts but cannot be applied to quantify those compounds. Based on the results of extracting endogenous extracts with the seven other solvents, dichloromethane was selected as the solvent for subsequent endogenous extract determination experiments.

### 2.3. Determination Results of Endogenous Extracts in Flowers of Seven Lily Cultivars

We used dichloromethane to extract the petals of the other six lily cultivars except for *Lilium* ‘Viviana’ during their peak flowering stage to determine the types and contents of endogenous extracts in the petals. The results are shown in Table 2 (the endogenous extract determination results for *Lilium* ‘Viviana’ flowers are the same as in Table S2). 

We found that eighteen endogenous extracts were detected in the petals of seven lily cultivars, and more types of compounds were extracted from the four richly fragrant lily cultivars (‘Palazzo’, ‘Pink News’, ‘Tiber’ and ‘Viviana’)—we extracted fifteen, twelve, eleven and eleven compounds, respectively. We also extracted two, six and one compounds from the three lightly or almost non-fragrant lily cultivars (‘Trebbiano’, ‘Avalon Sunset’ and ‘Tresor’). Regarding the compositions of the compounds, methyl benzoate, ethyl benzoate and 2-methoxy-4-vinylphenol were detected in six cultivars; creosol, geranylacetone and farnesol were detected in five cultivars; β-ocimene, linalool and isoeugenol were detected in four cultivars; eugenol was detected in three cultivars; 1,8-cineole and α-terpineol were only detected in the endogenous extracts of ‘Pink News’ and ‘Palazzo’; 4-methylveratrole, geraniol and isovanillin were only present in ‘Pink News’, ‘Viviana’ and ‘Tiber’, respectively; benzoic acid, benzyl benzoate and benzyl salicylate were only present in ‘Palazzo’. 

**Figure 2:**
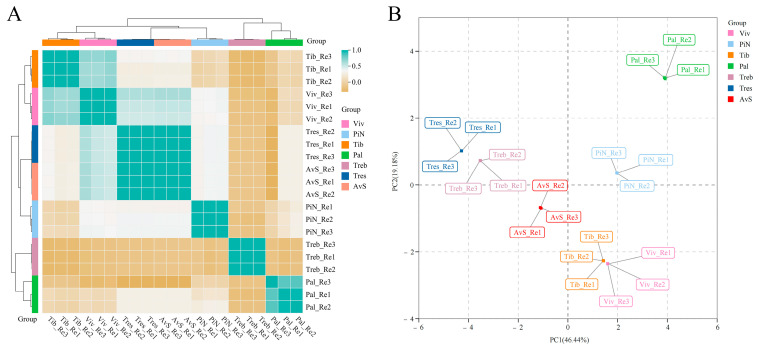
Sample correlation analysis (**A**) and PCA (**B**) of endogenous extracts from petals of seven lily cultivars. AvS: ‘Avalon Sunset’; Pal: ‘Palazzo’; PiN: ‘Pink News’; Tib: ‘Tiber’; Treb: ‘Trebbiano’; Tres: ‘Tresor’; Viv: ‘Viviana’. Three biological replicates were performed for the endogenous extract determination of each cultivar.

**Figure 3:**
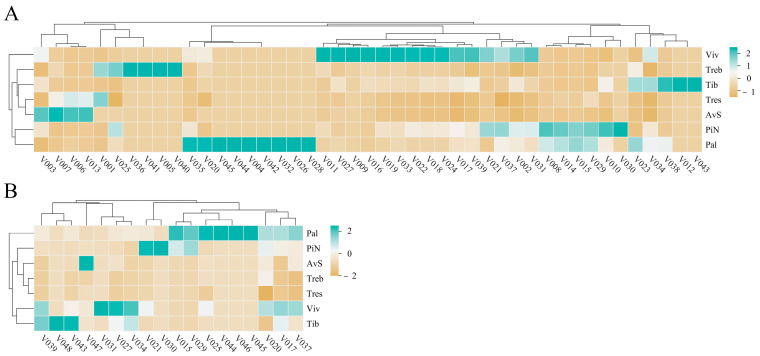
Heat map of contents of VOCs (**A**) and endogenous extracts (**B**) in petals of seven lily cultivars during the peak flowering stage. AvS: ‘Avalon Sunset’; Pal: ‘Palazzo’; PiN: ‘Pink News’; Tib: ‘Tiber’; Treb: ‘Trebbiano’; Tres: ‘Tresor’; Viv: ‘Viviana’. The detailed compounds (VOCs and endogenous extracts) information in Table 1 and Table 2.

We also conducted sample correlation analyses and PCA using the measurement results of endogenous extracts in the petals of seven lily cultivars, and the results are shown in Figure 2. The determination results of endogenous extracts in seven cultivars show good repeatability. Based on the results of the sample correlation analysis, the correlations between ‘Tiber’ and ‘Viviana’; ‘Tresor’, ‘Avalon Sunset’ and ‘Pink News’; and ‘Trebbiano’ and ‘Palazzo’ were the closest. According to the PCA results, four richly fragrant lily cultivars (‘Palazzo’, ‘Pink News’, ‘Tiber’ and ‘Viviana’) were separated from the other three lightly or almost non-fragrant lily cultivars (‘Trebbiano’, ‘Avalon Sunset’ and ‘Tresor’) in the first principal component (PC1 46.44%). This result is similar to the PCA results of VOCs in seven cultivars (Figure 1B). Figure 3 is the heat map of the contents of VOCs and endogenous extracts in the petals of seven lily cultivars during the peak flowering stage.

### 2.4. The Results of Linear Regression Analysis between Emission Ratios and Boiling Points of Compounds in Four Lily Cultivars

**Figure 4:**
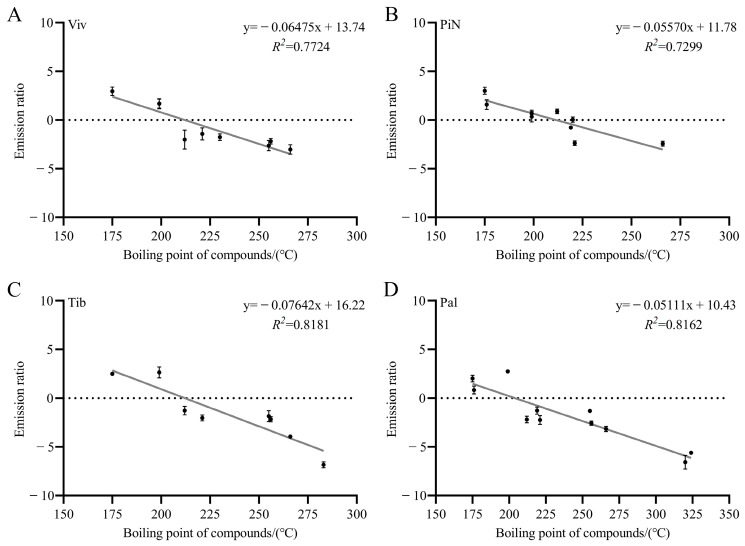
Linear regression analysis results between emission ratios and boiling points of four lily cultivars. (**A**) ‘Viviana’; (**B**) ‘Pink News’; (**C**) ‘Tiber’; (**D**) ‘Palazzo’. The emission ratios and boiling points of compounds are shown in Table S4.

To observe the emission status of each compound in petals more intuitively, we used the natural logarithm of the ratio of VOC content to endogenous extract contents to represent the emission ratio of volatile substance [19]. The emission ratios of volatile substances are shown in Table S4. The extent of emission ratio is related to the difficulty of the compound’s volatilization. At the same time, to investigate whether the emission ratios of VOCs were related to their boiling points, we selected four richly fragrant lily cultivars and conducted linear regression analyses between the emission ratios of compounds and boiling points. The results are shown in Figure 4; we found that the coefficients of regression (*R*^2^) between emission ratios and boiling points in four cultivars were all greater than 0.7, with the *R*^2^ of ‘Tiber’ and ‘Palazzo’ greater than 0.8. Therefore, it could be considered that there were linear negative correlations between the emission ratios and boiling points in four cultivars, indicating downward trends in emission ratios as the boiling points of compounds increased. The linear regression equation for ‘Viviana’ is y = −0.06475x + 13.74 (*R*^2^ = 0.7724); that for ‘Pink News’ is y = −0.05570x + 11.78 (*R*^2^ = 0.7299), that for ‘Tiber’ is y = −0.07642x + 16.22 (*R*^2^ = 0.8181), and that for ‘Palazzo’ is y = −0.05111x + 10.43 (*R*^2^ = 0.8162).

### 2.5. The Results of qRT-PCR and Correlation Analysis of TPS and BSMT Genes in Seven Lily Cultivars

**Figure 5:**
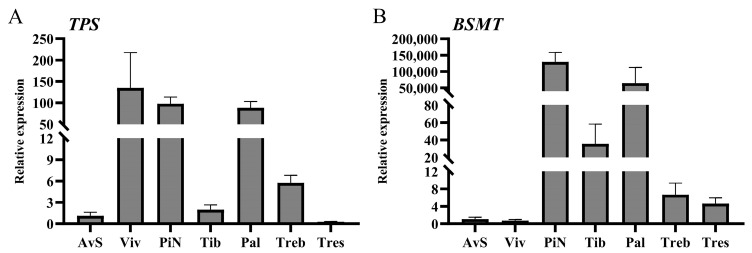
Expressions of *TPS* (**A**) and *BSMT* (**B**) in the petals of seven cultivars during the peak flowering stage. AvS: ‘Avalon Sunset’; Pal: ‘Palazzo’; PiN: ‘Pink News’; Tib: ‘Tiber’; Treb: ‘Trebbiano’; Tres: ‘Tresor’; Viv: ‘Viviana’.

The qRT-PCR analyses were conducted to clarify the expressions of *TPS* and *BSMT* in the petals of seven lily cultivars. Terpene synthase (TPS) is a downstream key enzyme in the terpenoid biosynthesis pathway, directly related to terpenoid biosynthesis. Benzoic acid/salicylic acid carboxyl methyltransferase (BSMT) can catalyze the generation of methyl benzoate or methyl salicylate from benzoic acid or salicylic acid. The *TPS* and *BSMT* employed in this study were related to the biosynthesis of monoterpenoids (linalool and β-ocimene) and methyl benzoate, which have been confirmed to be present in *Lilium*. The results of qRT-PCR are shown in Figure 5. The *TPS* had the lowest expression level in ‘Tresor’ and ‘Avalon Sunset’, followed by ‘Tiber’ and ‘Trebbiano’, and it was highly expressed in ‘Palazzo’, ‘Pink News’ and ‘Viviana’, especially in ‘Viviana’ (134.879). *BSMT* had the highest expression level in ‘Pink News’ (129,544.389), followed by ‘Palazzo’ (64,054.895), and the *BSMT* expression levels of these two cultivars reached over 50,000, far higher than the other cultivars, followed by ‘Tiber’, ‘Trebbiano’, ‘Tresor’ and ‘Avalon Sunset’. Surprisingly, the *BSMT* expression level in ‘Viviana’ was the lowest among these seven cultivars, forming a massive contrast with the *TPS* expression level.

**Figure 6:**
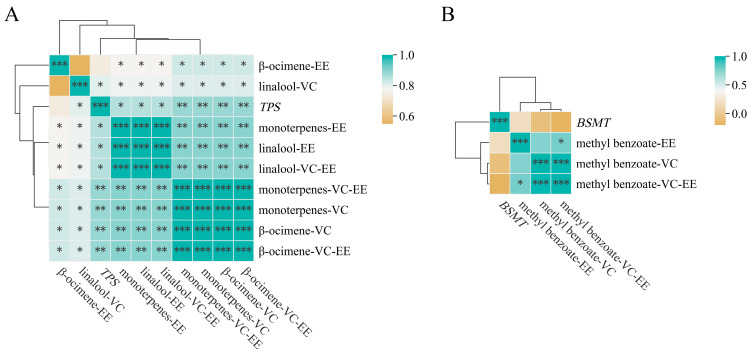
Correlation analysis results between the expression levels of two genes and the contents of compounds. (**A**) Correlation analysis between *TPS* expression level and corresponding compound content, “VC” refers to the content of VOC, “EE” refers to the content of endogenous extract, “VC-EE” refers to the total content (VOC + endogenous extract), “monoterpenes” refers to linalool + β-ocimene. (**B**) Correlation analysis between *BSMT* expression level and corresponding compound content, “VC” refers to the content of VOC, “EE” refers to the content of endogenous extract, “VC-EE” refers to the total content (VOC + endogenous extract). “*”, “**” and “***” respectively represent the significant correlation at the 0.05, 0.01 and 0.001 levels (double-tailed).

We conducted the correlation analyses based on the relative expression levels of *TPS* and *BSMT* in the petals of seven lily cultivars combined with the contents of linalool, β-ocimene, monoterpenes (linalool + β-ocimene) and methyl benzoate (including VOC content, endogenous extract content and total contents (VOCs + endogenous extracts)) in the petals of seven lily cultivars. The results are shown in Figure 6 and Table S5. In Figure 6A, except for the correlation between *TPS* expression level and the endogenous content of β-ocimene, and the correlation between the volatile content of linalool and the endogenous content of β-ocimene, which were not significant, there was a significant correlation between all other combinations, and the correlation coefficients were all greater than 0.7. In Figure 6B, the expression level of *BSMT* is not significantly correlated with the content of methyl benzoate (including volatile, endogenous and total methyl benzoate contents), and there is no significant positive correlation (correlation is 0.75) between the volatile and endogenous contents of methyl benzoate. However, there is a significant positive correlation between the volatile content and the total content of methyl benzoate, and between the endogenous content and the total content of methyl benzoate.

## 3. Discussion

Through the analysis of the types and contents of VOCs in seven cultivars of lily flower petals, we can confirm that the main VOCs in lily flower petals are terpenoids and phenylpropanoids/benzenoids. The most important group of compounds is terpenoids, especially monoterpenoids, which account for more than half of the total number of terpenoid VOCs. Monoterpenoids such as linalool, β-ocimene, 1,8-cineole and so on are also commonly present, with high contents, in the petals of many richly fragrant lily cultivars. Besides the terpenoid VOCs, the contents of phenylpropanoid/benzenoid VOCs in lily petals cannot be ignored, while methyl benzoate and ethyl benzoate are also present in most lily cultivars. The content of methyl benzoate is much higher than that of ethyl benzoate, and is even higher than some high-content monoterpenoid VOCs. Therefore, methyl benzoate is the most critical VOC of phenylpropanoids/benzenoids in lily flower petals, and it is also one of the most essential lily fragrance components, just like linalool, β-ocimene and 1,8-cineole. Our research conclusion is consistent with previous research [11]. Floral fragrance is crucial for lily reproduction and can attract insect pollination [22]; methyl benzoate has a fruity odor, is present in the flowers of many plants, and plays a vital role in attracting insects for pollination [23].

In this study, we also found that the phenylpropanoid/benzenoid VOCs in ‘Palazzo’ (OT hybrid) petals were more plentiful than other cultivars, such as methyl phenylacetate, methyl salicylate, butyl benzoate, benzyl benzoate and benzyl salicylate, which were only detected in the VOCs of ‘Palazzo’ flower petals, although these components are not plentiful. OT hybrid lilies originated from hybridization between Oriental and Trumpet hybrid lilies, and the VOCs of lily flowers can be traced back to their parents [11]. Therefore, many phenylpropanoid/benzenoid VOCs detected in ‘Palazzo’ flower petals are likely from their parents and provide OT lily flowers with rich and diverse phenylpropanoid/benzenoid VOCs. Improving the fragrance compositions of lily flowers can be achieved through conventional hybridization methods, thereby allowing the cultivation lily cultivars with bright colors, diverse fragrances and unique flower morphologies. 

There are many lily cultivars worldwide, and they have various floral fragrance types, ranging from weak-scented to strong-scented. Du et al. measured many lily cultivar petals’ VOCs; they classified the mean scent compounds into five groups (herbal, fruity, cool, floral, and spicy) based on their odor description, and the scents of cultivars were classified into six groups based on the ratios of total amounts of scent compounds for each group [24]. In this study, we selected different types of lily cultivars based on the intensity of their odors (richly fragrant, lightly fragrant and almost non-fragrant). The differences in VOC contents and abundances are the basis for the differences in lily fragrance [25], and the wide diversity of lily fragrance makes lily a rich biochemical and genetic resource for fragrance. In addition, similar to floral fragrance, flower color is also vital for plant reproduction. Studies have shown that there may be a competitive relationship between pigments (such as flavonoids) and floral aroma components (phenylpropanoids/benzenoids) in plant flower biosynthesis, as they all belong to the phenylpropanoid metabolism pathway [26]. Therefore, some lily cultivars (such as Asiatic hybrids) have abundant colors but lack fragrance, and some lily cultivars (such as Oriental hybrids, Longiflorum hybrids and OT hybrids) have strong floral fragrances but only one color.

We used eight solvents to extract the endogenous extracts from the petals of the Oriental lily ‘Viviana’ at the peak flowering stage, and we conducted qualitative and quantitative analyses of the endogenous extracts to find the most suitable extraction solvent. The polarity of these eight solvents varies, ranging from solvents with low polarity (such as petroleum ether and n-hexane) to those with high polarity (such as methanol and ethanol). Based on the results of this study, we found that benzene and dichloromethane could extract more endogenous extracts, making them suitable solvents for extracting the endogenous volatile components in lily petals. The extraction effects of other solvents with smaller or medium polarity (petroleum ether, n-hexane, and ethyl acetate) were also relatively good. However, three alcohol solvents with greater polarity (methanol, ethanol and n-butanol) were unsuitable for extracting endogenous volatile components from lily petals. There are many studies on the extraction of endogenous volatile components from flower petals using organic solvents such as ethyl acetate [21,27,28,29,30,31], dichloromethane [20,32,33], petroleum ether [34], pentane [35,36] and n-hexane [37]. The results of using different solvents for endogenous volatile component extractions are also different [38,39]; the most suitable organic solvent can be selected for different sample materials. This experiment provides a reference for exploring and improving the extraction methods of endogenous volatile components from the petals of lilies and other plants from the perspective of solvent selection. 

According to the determination results of endogenous extracts, the numbers of endogenous extracts in richly fragrant lily cultivars were higher than those in lightly fragrant and almost non-fragrant lily cultivars; this finding is similar to the determination results of VOCs. However, from the calculation results of emission ratios for each compound (Table S4), we have learned that compounds with high boiling points often have lower emission ratios. Therefore, high-boiling-point compounds are more difficult to discharge to the outside. According to Figure 6, we can see almost linear negative correlations between the emission ratios and boiling points of compounds from lily petals, consistent with the research results of Kondo et al. [19]. According to the results in Table S4, there are slight differences in the emission ratios of the same compounds between different cultivars, such as methyl benzoate and ethyl benzoate in ‘Pink News’. This result might be due to the influence of transporters related to the transportation of VOCs. It has been confirmed that multiple VOCs were transported from the intracellular region to the extracellular region (external environment) through ABC transporters (ABCG transporters), such as phenylpropanoids/benzenoids (benzyl alcohol and methyl benzoate) [40], monoterpenoid (geraniol) [37] and sesquiterpenoid (β-caryophyllene) [41]. The release of these VOCs was related to ABC (ABCG) transporters, and the overexpression of ABC (ABCG) transporter genes could lead to an increase in VOCs emissions (to the outside), resulting in a decrease in intracellular compound contents, thus causing changes in emission ratios. Further exploration is needed into the mechanism of ABC transport in the transportation of VOCs of lilies in the future.

The qRT-PCR experiments of *TPS* and *BSMT* were conducted on the petals of seven lily cultivars. It was found that the expression level of *TPS* in ‘Tiber’ was relatively low (relative expression level was only 1.967), even lower than that in ‘Trebbiano’ (relative expression level was 5.729). However the degrees of release of the main monoterpenoid VOCs (β-ocimene and linalool) in ‘Tiber’ petals were much higher than those in ‘Trebbiano’. Zhang et al. [14] screened three *TPS* from the RNA-seq data of petals at different flowering stages of the Oriental lily ‘Siberia’, but the expression levels of these three genes differed in ‘Siberia’. *LiTPS2* had the highest expression level among these three genes and played the most crucial role in the biosynthesis of monoterpenoid VOCs (β-ocimene and linalool). Similarly, Abbas et al. [4,13] also screened multiple *TPS* in the Oriental lily ‘Siberia’. We speculated that the *TPS* used for the qRT-PCR in this study (*LiTPS2*) might not be the primary gene in ‘Tiber’. In the same way, the expression levels of *BSMT* in ‘Viviana’ and ‘Tiber’ were also low, but in reality, they showed higher emissions and endogenous contents of methyl benzoate. Besides *BSMT*, benzoic acid carboxyl methyltransferase (BAMT) can also catalyze the production of methyl benzoate from benzoic acid, which has been found in many plants [42,43]. Additionally, there may be more than one *BSMT* in a plant; *HcBSMT1* and *HcBSMT2* are simultaneously present in *Hedychium coronarium*, and both *HcBSMT1* and *HcBSMT2* can catalyze benzoic acid or salicylic acid to produce methyl benzoate or methyl salicylate [44]. Therefore, there are likely other types of *BAMTs* or *BSMTs* that are related to the synthesis of methyl benzoate in ‘Viviana’ and ‘Tiber’. In addition, a study has shown that a BAHD acyltransferase gene *LoAAT1* also contributes to the biosynthesis of methyl benzoate in the petals of *Lilium* ‘Siberia’; the differential expression of this gene in different lily cultivars may also be a reason for the differences in methyl benzoate content among these cultivars [45]. We conducted correlation analyses between the relative expression levels of two genes (*TPS* and *BSMT*) in the petals of seven lily cultivars and the contents of VOCs and endogenous extracts (β-ocimene, linalool and methyl benzoate). We found almost exclusively strong positive correlations between the *TPS* expression level and the contents of monoterpenoids (VOCs and endogenous extracts) in seven cultivars, which also proves that *LiTPS2* was crucial for the synthesis of monoterpenoids in lilies. However, there were no correlations between the expression levels of *BSMT* and the contents of methyl benzoate (VOCs and endogenous extracts) in seven cultivars. The reason for this result might be similar to that given above, which is that the genes with similar functions (*BSMT* and *BAMT*) had diversity.

## 4. Materials and Methods

### 4.1. Plant Materials

The plant materials were the flowers of seven lily cultivars. These flowers have varying degrees of fragrance and come from five different hybrid groups. Table 3 shows the cultivar information, flower images and fragrance descriptions of seven lily cultivars. All lily cultivars were sourced from the Netherlands and the bulbs were purchased from Kunming Sailan Trading Co., Ltd. (Kunming, China). We harvested the lilies’ flowers before blooming and performed regular nurturing procedures in the illuminating incubator for experimentation.

### 4.2. Instruments and Reagents

The SPME assembly 50/30 µm divinylbenzene/carboxyl/polydimethylsiloxane (DVB/CAR/PDMS) (Supelco, 57328-U) was bought from Merck Chemicals (Shanghai) Co., Ltd. (Shanghai, China). The standard of the n-alkane mixture (C7–C40) and ethyl decanoate standard (CAS: 110-38-3) were bought from Shanghai yuanye Bio-Technology Co., Ltd. (Shanghai, China). Organic solvents of HPLC grade (ethyl acetate, dichloromethane, benzene, n-hexane, petroleum ether, methanol, ethanol and n-butanol) were bought from Concord Technology (Tianjin) Co., Ltd. (Tianjin, China).

### 4.3. Collection and Determination of Lily Flower Scents

To collect VOCs, we employed HS-SPME as described in the previous study [46] and made some modifications. After placing the flowers in the container, 5 μL of standard solution (methanol solution of ethyl decanoate, *v*/*v* 1:1000) was added to the inner side of the container and then we sealed the container. We extracted VOCs from each flower sample at 40 °C for 30 min using fibers. After extraction, the fibers were desorbed for 5 min at 250 °C in the inlet and then GC/MS analysis was performed. GC/MS was carried out using Agilent 7890B GC-5977A MSD (Agilent, Santa Clara, CA, USA) equipped with DB-5MS (Agilent, Santa Clara, CA, USA) chromatographic column (30 m × 0.25 mm × 0.25 μm); the Agilent 7890B GC-5977A MSD was calibrated and tested. A sample containing 1 pg of octafluoronaphthalene (313-72-4) was injected into GC-MS for analysis and this was repeated six times, with the signal-to-noise value of 222, the Peak Area Repeatability of 5.6%, and the Retention Time Repeatability of 0.0%. All calibration items met the technical requirements. The chromatography program started at 50 °C, which was maintained for 4 min, then the temperature was increased to 270 °C at a rate of 10 °C/min and held at this temperature for 5 min; the carrier gas was helium. The parameters for the mass spectrometer were that the ionization mode of MS was EI, the electron energy was 70 eV, the scanning range of mass to charge ratio (*m*/*z*) was 30~500, the ion source temperature was 220 °C, and the interface temperature of GC/MS was 250 °C. 

The VOCs were analyzed using the Agilent MassHunter Qualitative Analysis B.06.00 software. The National Institute of Standards and Technology 08 library and Kovats Retention Index (RI) were employed to identify the VOCs, and the RIs of compounds were calculated by measuring the C7–C40 n-alkane standard. The content of each compound was calculated based on the ratio of the compound peak area to the standard peak area. 

The calculation of RI was achieved using the Kovats Retention Index calculator (https://www.pherobase.com/kovats/ (accessed on 12 August 2023)) [47]. The formula for calculating the RI is as follows [48]:

$$I=100\times \left(n+\frac{{RT}_{r\left(x\right)}-{RT}_{r\left(n\right)}}{{RT}_{r\left(N\right)}-{RT}_{r\left(n\right)}}\right)$$

*I*: RI of the compound to be measured; *n*: the number of carbon atoms of the smaller neighboring alkane; *N*: the number of carbon atoms of the bigger neighboring alkane; *RT_r_*_(*x*)_: retention time of the compound to be measured; *RT_r_*_(*n*)_: retention time of the smaller neighboring alkane; *RT_r_*_(*N*)_: retention time of the bigger neighboring alkane.

### 4.4. Extraction and Determination of Endogenous Extracts from Lily Flowers

The extraction of endogenous extracts from lily flowers was achieved using the OSE method. We also used methods from a previous study [37] and made some modifications. We weighed about 2 g of petals during the peak flowering stage and cut them into strips, placing them in a 15 mL centrifuge tube. We added 10 mL organic solvent and shook the centrifuge tube at the usual temperature for 24 h to extract the endogenous extracts from lily petals. After extraction, we centrifuged the sample at 4000× *g* rpm for 10 min and used the 0.22 μm organic filter for filtration. We utilized a nitrogen blowing instrument to concentrate the extract to 1 mL, adding 5 μL of standard solution for standby.

The endogenous extract samples were injected into GC using the automatic sampler and the injection volume was 1 μL. The GC and MS conditions for determining endogenous extracts and performing the qualitative analysis of compounds were the same as those for VOCs.

### 4.5. Extraction of Total RNA and Synthesis of cDNA

The extraction of total RNA and synthesis of cDNA of lily petals from different cultivars was done via the methods used by our research group previously [17]. We separately used a total RNA extraction kit (Omega, GA, USA) and PrimeScript^TM^ RT Reagent Kit with gDNA Eraser (Perfect Real Time) (TaKaRa, Shiga, Japan). RNA quality and concentration were analyzed using a NanoDrop micro nucleic acid assay and 1% gel electrophoresis, and the generated cDNA was stored at −20 °C for the following qRT-PCR.

### 4.6. Gene Expression Analysis of TPS and BSMT

To find out the expression levels of *TPS* and *BSMT* in different cultivars of petals, the PikoReal real-time PCR system (Thermo Fisher Scientific, Waltham, MA, USA) and TB Green^®^ Premix Ex TaqTM II (Tli RNaseH Plus) (TaKaRa, Shiga, Japan) were used for qRT-PCR [17]. The primers used in qRT-PCR are shown in Table S6 and *Actin* was selected as the internal reference gene for each sample (GenBank: JX826390) [49]. The expression levels of both genes in ‘Avalon Sunset’ were set to 1.

### 4.7. Statistical Analysis and Picture Drawing

The sample correlation heat map, PCA diagram, cluster heat map and correlation heat map were completed using the Metware Cloud (https://cloud.metware.cn, accessed on 17 September 2023). GraphPad Prism 8 software was utilized to draw the column chart and linear regression analysis. SPSS 26 software was utilized for correlation analyses between the expression levels of two genes (*TPS* and *BSMT*) and the contents of compounds. The boiling points of the compounds were referenced in ChemSrc (https://www.chemsrc.com/casindex/ (accessed on 28 August 2023)), Molbase Chemical Dictionary (https://www.molbase.cn/ (accessed on 28 August 2023)) and PubChem (https://pubchem.ncbi.nlm.nih.gov/ (accessed on 28 August 2023)).

## 5. Conclusions

This study employed the HS-SPME-GC-MS, OSE-GC-MS and qRT-PCR technologies to discover the VOCs, endogenous extracts and *TPS* and *BSMT* expression levels in the petals of seven lily cultivars. We also conducted linear regression analyses between the emissions ratios and boiling points of compounds, and conducted correlation analyses based on the relative expression levels of *TPS* and *BSMT* combined with the contents of corresponding compounds in the petals of seven lily cultivars. The following conclusions were drawn:

(1) Forty-five kinds of VOCs were detected in the petals of seven lily cultivars. The main VOCs were monoterpenoids (β-ocimene, linalool, 1,8-cineole) and phenylpropanoids/benzenoids (methyl benzoate);
(2) Dichloromethane was the most suitable for extracting the endogenous extracts from the petals of *Lilium* ‘Viviana’ among the eight commonly used organic solvents;
(3) When using dichloromethane as the solvent, eighteen kinds of endogenous extracts were detected in the petals of seven lily cultivars;
(4) All linear correlations were negative and significant between the emission ratios and boiling points of VOCs in the petals of the four lily cultivars;
(5) *TPS* was highly expressed in ‘Viviana’, ‘Pink News’ and ‘Palazzo’, and *BSMT* was highly expressed in ‘Pink News’ and ‘Palazzo’ in the petals of the seven lily cultivars. Almost all correlations were strong and positive between the expression levels of *TPS* and the contents of monoterpenoids (linalool, β-ocimene and linalool + β-ocimene), and there were no correlations between the expression levels of *BSMT* and the contents of methyl benzoate in the seven cultivars.

## Figures and Tables

**Table 1:** Determination of volatile organic compounds (VOCs) in the petals of seven lily cultivars during the peak flowering stage.

No.	Compounds	CAS	Mea RI	Lite RI	Compound Content ng·g^−1^·h^−1^ (Mean ± SE)						
					Viv	PiN	Tib	Pal	Treb	Tres	AvS
terpenoid											
V008	α-pinene	80-56-8	938	939	-	439.14 ± 114.23	-	267.08 ± 48.39	-	-	-
V009	β-myrcene	123-35-3	989	988	3398.10 ± 1045.07	-	-	-	-	-	-
V010	β-pinene	127-91-3	989	989	-	2060.84 ± 536.45	922.60 ± 100.48	806.04 ± 172.36	337.00 ± 110.80	148.45 ± 10.27	-
V011	3-carene	13466-78-9	1006	1009	2980.53 ± 753.06	385.21 ± 72.61	-	133.40 ± 27.86	363.68 ± 103.96	-	-
V014	limonene	138-86-3	1031	1031	-	726.55 ± 187.21	-	535.54 ± 85.79	-	59.98 ± 6.03	-
V015	1,8-cineole	470-82-6	1039	1038	-	4323.14 ± 1097.69	-	4045.72 ± 926.27	-	249.70 ± 6.74	-
V016	trans-β-ocimene	3779-61-1	1039	1042	2404.50 ± 713.24	-	342.87 ± 60.09	-	203.32 ± 48.26	-	-
V017	β-ocimene	13877-91-3	1048	1048	13,229.08 ± 3292.51	6564.96 ± 1251.59	5192.03 ± 545.72	4451.70 ± 545.60	1993.95 ± 568.57	-	-
V018	γ-terpinene	99-85-4	1060	1062	949.32 ± 255.61	315.75 ± 79.27	259.78 ± 30.87	140.84 ± 46.44	119.78 ± 24.71	-	-
V019	terpinolene	586-62-9	1083	1088	920.11 ± 264.05	227.54 ± 58.21	168.90 ± 29.29	90.00 ± 22.50	97.89 ± 7.75	-	-
V021	linalool	78-70-6	1104	1103	4491.74 ± 1369.78	4114.53 ± 776.87	-	-	-	-	-
V022	allo-ocimene	673-84-7	1130	1129	4167.40 ± 1137.73	1501.23 ± 340.77	760.39 ± 89.50	545.98 ± 124.39	433.12 ± 118.28	-	-
V023	cosmene	460-01-5	1135	1130	-	-	148.97 ± 22.10	157.48 ± 18.35	81.58 ± 19.52	-	-
V024	neo-allo-ocimene	7216-56-0	1143	1131	4012.35 ± 1066.74	1340.63 ± 330.76	1013.28 ± 129.11	578.55 ± 179.21	473.62 ± 88.65	-	-
V029	α-terpineol	98-55-5	1203	1200	-	165.15 ± 4.81	-	131.53 ± 22.60	-	40.84 ± 6.95	-
V031	geraniol	106-24-1	1253	1255	51.28 ± 14.13	31.30 ± 4.02	9.61 ± 1.68	14.39 ± 0.52	-	-	-
V036	β-caryophyllene	87-44-5	1432	1420	-	-	-	-	617.07 ± 127.01	-	-
V037	geranylacetone	689-67-8	1451	1460	61.93 ± 11.52	67.03 ± 11.99	23.46 ± 1.39	43.26 ± 3.22	18.29 ± 1.24	7.70 ± 1.15	16.68 ± 3.39
V038	cis-β-farnesene	28973-97-9	1453	1458	-	-	6.05 ± 0.58	2.82 ± 0.40	-	-	-
V040	α-caryophyllene	6753-98-6	1471	1463	-	-	-	-	57.20 ± 13.77	-	-
V041	β-ionone	14901-07-6	1485	1486	-	-	-	-	13.63 ± 2.92	-	-
V042	α-farnesene	502-61-4	1506	1508	-	-	-	34.97 ± 8.83	-	-	-
V043	farnesol	4602-84-0	1720	1722	-	-	2.95 ± 0.47	-	-	-	-
phenylpropanoid/benzenoid compound											
V020	methyl benzoate	93-58-3	1099	1096	2099.60 ± 799.42	387.75 ± 53.65	1011.49 ± 212.38	5462.20 ± 464.29	1241.80 ± 203.36	-	937.92 ± 299.18
V025	ethyl benzoate	93-89-0	1176	1172	39.51 ± 21.23	140.69 ± 20.62	20.28 ± 0.87	62.63 ± 16.68	174.02 ± 22.50	-	48.63 ± 7.47
V026	methyl phenylacetate	101-41-7	1177	1177	-	-	-	19.74 ± 1.10	-	-	-
V027	creosol	93-51-6	1194	1193	429.85 ± 144.03	16.85 ± 3.43	89.84 ± 16.17	43.16 ± 10.47	-	-	25.87 ± 6.40
V028	methyl salicylate	119-36-8	1200	1198	-	-	-	40.72 ± 10.02	-	-	-
V030	4-methylveratrole	494-99-5	1240	1230	-	206.17 ± 15.27	-	-	-	-	-
V032	phenethyl acetate	103-45-7	1257	1258	-	-	-	27.36 ± 3.13	-	-	-
V034	eugenol	97-53-0	1363	1356	20.75 ± 6.87	12.82 ± 1.23	25.17 ± 4.65	17.95 ± 1.59	-	-	-
V035	butyl benzoate	136-60-7	1380	1377	12.27 ± 3.97	10.96 ± 1.20	-	39.90 ± 4.76	-	-	-
V039	isoeugenol	97-54-1	1456	1460	95.86 ± 27.24	34.14 ± 3.51	40.11 ± 5.21	29.29 ± 2.87	-	-	-
V044	benzyl benzoate	120-51-4	1779	1775	-	-	-	2.56 ± 0.13	-	-	-
V045	benzyl salicylate	118-58-1	1883	1876	-	-	-	1.60 ± 0.45	-	-	-
alcohol											
V002	3-hexen-1-ol	544-12-7	859	856	45.50 ± 10.75	31.06 ± 10.71	20.04 ± 3.25	21.18 ± 7.01	-	-	-
V004	cis-2-hexen-1-ol	928-94-9	872	872	-	-	-	90.43 ± 10.39	-	-	-
V005	trans-2-hexen-1-ol	928-95-0	872	874	-	-	-	-	453.50 ± 107.69	-	-
ether											
V006	dibutyl ether	142-96-1	884	888	-	-	-	60.17 ± 14.49	-	631.57 ± 101.67	1092.70 ± 206.84
aldehyde											
V001	trans-2-hexanal	6728-26-3	859	854	-	-	-	-	1667.71 ± 322.39	1790.20 ± 417.91	264.85 ± 53.70
ester											
V003	methyl tiglate	6622-76-0	874	876	631.37 ± 267.91	409.93 ± 112.67	392.34 ± 39.50	-	-	-	1153.48 ± 216.88
V007	butyl acrylate	141-32-2	893	902	239.05 ± 66.72	108.81 ± 12.84	178.67 ± 30.55	132.99 ± 15.07	402.46 ± 123.10	852.47 ± 262.69	2064.26 ± 377.97
V012	trans-3-hexen-1-ol acetate	3681-82-1	1008	1005	-	-	1870.83 ± 93.01	-	-	-	-
V013	trans-2-hexenyl acetate	2497-18-9	1014	1014	-	-	-	-	-	58.24 ± 14.13	109.76 ± 18.24
V033	methyl decanoate	110-42-9	1323	1324	71.43 ± 23.45	31.13 ± 4.34	28.71 ± 1.60	28.34 ± 7.55	26.52 ± 8.23	18.90 ± 0.51	18.85 ± 1.69

The “-” in the table indicates that this compound has not been detected or its content is low. Kovats Retention Index (RI) can be divided into measured RI (Mea RI) and literature RI (Lite RI), where the Mea RI was calculated according to the formula in Section 4.3 and the Lite RI was obtained from the NIST Standard Reference Database Number 69 (https://webbook.nist.gov/chemistry/ (accessed on 25 August 2023)).

**Table 2:** Determination of endogenous extracts in petals of seven lily cultivars during the peak flowering stage.

No.	Compounds	CAS	Mea RI	Lite RI	Compound Contents ng·g^−1^ (mean ± SE)						
					Viv	PiN	Tib	Pal	Treb	Tres	AvS
V015	1,8-cineole	470-82-6	1039	1038	-	811.72 ± 13.10	-	1674.00 ± 40.68	-	-	-
V017	β-ocimene	13877-91-3	1048	1048	644.11 ± 0.90	308.60 ± 2.90	419.22 ± 12.22	578.65 ± 41.99	-	-	-
V020	methyl benzoate	93-58-3	1099	1096	344.58 ± 21.01	278.62 ± 45.41	70.75 ± 9.55	350.02 ± 9.74	231.37 ± 15.98	-	203.24 ± 12.66
V021	linalool	78-70-6	1104	1103	759.80 ± 23.19	2007.47 ± 50.59	72.58 ± 9.15	364.22 ± 16.46	-	-	-
V046	benzoic acid	65-85-0	1172	1170	-	-	-	834.96 ± 85.51	-	-	-
V025	ethyl benzoate	93-89-0	1176	1172	221.22 ± 9.04	56.86 ± 4.80	78.53 ± 22.78	519.41 ± 49.22	87.37 ± 16.31	-	92.71 ± 19.47
V027	creosol	93-51-6	1194	1193	1599.97 ± 24.99	175.03 ± 15.89	658.38 ± 13.41	381.68 ± 14.16	-	-	293.89 ± 21.30
V029	α-terpineol	98-55-5	1203	1200	-	358.13 ± 21.69	-	456.36 ± 20.24	-	-	-
V030	4-methylveratrole	494-99-5	1240	1230	-	202.33 ± 31.16	-	-	-	-	-
V031	geraniol	106-24-1	1253	1255	282.28 ± 32.64	-	-	-	-	-	-
V047	2-methoxy-4-vinylphenol	7786-61-0	1323	1324	1962.82 ± 119.96	1142.59 ± 24.06	1493.06 ± 94.50	781.76 ± 332.74	-	941.27 ± 239.37	9703.41 ± 1053.98
V034	eugenol	97-53-0	1363	1356	258.50 ± 11.52	-	155.01 ± 22.10	66.69 ± 4.42	-	-	-
V048	isovanillin	621-59-0	1413	1401	-	-	683.86 ± 60.84	-	-	-	-
V037	geranylacetone	689-67-8	1451	1460	523.81 ± 29.45	256.68 ± 42.15	210.38 ± 43.92	559.23 ± 28.23	-	-	251.32 ± 36.13
V039	isoeugenol	97-54-1	1456	1460	1809.00 ± 36.64	385.34 ± 16.81	2028.74 ± 60.16	689.01 ± 68.06	-	-	-
V043	farnesol	4602-84-0	1720	1722	821.28 ± 26.94	239.73 ± 35.69	2698.67 ± 6.82	563.89 ± 7.21	-	-	208.70 ± 13.68
V044	benzyl benzoate	120-51-4	1779	1775	-	-	-	701.87 ± 45.88	-	-	-
V045	benzyl salicylate	118-58-1	1883	1876	-	-	-	1048.62 ± 84.77	-	-	-

The “-” in the table indicates that this compound has not been detected or its content is low. Kovats Retention Index (RI) could be divided into measured RI (Mea RI) and literature RI (Lite RI); the Mea RI was calculated according to the formula in Section 4.3 and the Lite RI was obtained from the NIST Standard Reference Database Number 69 (https://webbook.nist.gov/chemistry/ (accessed on 25 August 2023)).

**Table 3:** Information about the seven lily cultivars used in this study.

Cultivar	‘Avalon Sunset’	‘Palazzo’	‘Pink News’	‘Tiber’
Flower images	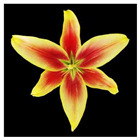	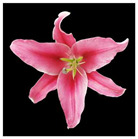	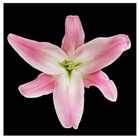	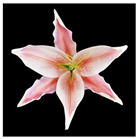
Abbreviation	AvS	Pal	PiN	Tib
Classification	OA	OT	O	O
Fragrance description	almost non-fragrant	richly fragrant	richly fragrant	richly fragrant
**Cultivar**	**‘Trebbiano’**	**‘Tresor’**	**‘Viviana’**	
Flower images	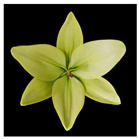	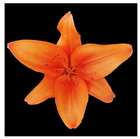	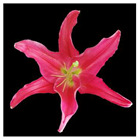	
Abbreviation	Treb	Tres	Viv	
Classification	LA	A	O	
Fragrance description	lightly fragrant	almost non-fragrant	richly fragrant	

Classification: A = Asiatic hybrid, LA = Longiflourm × Asiatic hybrid, O = Oriental hybrid, OA = Oriental × Asiatic hybrid, OT = Oriental × Trumpet hybrid.

## Data Availability

Data are contained within the article and Supplementary Materials.

## Supplementary Materials

The following supporting information can be downloaded at: https://www.mdpi.com/article/10.3390/molecules28247938/s1, Table S1: Retention time of C7–C40 n-alkanes. Table S2: Determination of endogenous extracts in petals of *Lilium* ‘Viviana’ during the peak flowering stage (excluding benzene). Table S3: The peak area of endogenous extracts from petals of *Lilium* ‘Viviana’ extracted by benzene. Table S4: Emission ratios of compounds in petals of seven lily cultivars during the peak flowering stage. Table S5: Correlation coefficients about the correlation analyses between the expression levels of two genes (*TPS* and *BSMT*) and the contents of compounds. Table S6: Primers used in qRT-PCR. Chromatograms: The chromatograms of all samples.
